# *Cryptosporidium parvum*: an emerging occupational zoonosis in Finland

**DOI:** 10.1186/s13028-023-00684-z

**Published:** 2023-06-22

**Authors:** Tuulia Enbom, Kristiina Suominen, Sirpa Laitinen, Jukka Ollgren, Tiina Autio, Ruska Rimhanen-Finne

**Affiliations:** 1 Animal Health Diagnostic Unit, Finnish Food Authority, Neulaniementie 4, Kuopio, Finland; 2 Department of Health Security, Finnish Institute for Health and Welfare, Mannerheimintie 166, Helsinki, Finland; 3 Occupational Safety, Finnish Institute of Occupational Health, Neulaniementie 4, Kuopio, Finland

**Keywords:** Biological Agents, Cryptosporidiosis, Gp60 subtypes, Occupational Health, Risk factors, Zoonoses

## Abstract

**Background:**

Cryptosporidiosis has increased in recent years in Finland. We aimed to identify risk factors for human cryptosporidiosis and to determine the significance of *Cryptosporidium parvum* as a causative agent. Based on notifications to the Finnish Infectious Disease Register (FIDR), we conducted a case-control study and genotyped *Cryptosporidium* species from patient samples from July to December 2019. We also retrieved the occupational cryptosporidiosis cases from 2011 to 2019 from the Finnish Register of Occupational Diseases (FROD).

**Results:**

Of 272 patient samples analyzed, 76% were *C. parvum* and 3% *C. hominis*. In the multivariable logistic regression analysis of 82 *C. parvum* cases and 218 controls, cryptosporidiosis was associated with cattle contact (OR 81, 95% confidence interval (CI) 26–251), having a family member with gastroenteritis (OR 34, 95% CI 6.2–186), and spending time at one’s own vacation home (OR 15, 95% CI 4.2–54). Of the cases, 65% had regular cattle contact. The most common gp60 subtypes identified were IIaA15G2R1 and IIaA13G2R1. In FROD, 68 recognized occupational cryptosporidiosis cases were registered in 2011–2019.

**Conclusions:**

*C. parvum* is the most common *Cryptosporidium* species found in humans in Finland and poses a moderate to high risk of occupational infection for people working with cattle. The number of occupational notifications of cryptosporidiosis increased between 2011 and 2019. Cryptosporidiosis should be recognized as an important occupational disease among persons working with livestock in Finland, criteria to identify occupational cryptosporidiosis need to be created, and occupational safety in cattle-related work should be improved.

## Background

Zoonoses are global concerns in the agricultural sector [1]. Zoonotic *Cryptosporidium parvum* is prevalent in cattle and humans worldwide. Cryptosporidiosis is a gastrointestinal zoonotic disease that may pose a risk of occupational illness to those working with cattle [2]. At least 44 *Cryptosporidium* species exist, of which *C. parvum* and *Cryptosporidium hominis* are the major species causing intestinal infection in humans [3]. Furthermore, *C. parvum* is the species of primary importance for cattle and causes diarrhea in young calves [4]. The infection is transmitted by the fecal–oral route among humans and animals, and between humans and animals.

Humans can contract *Cryptosporidium* infections from an oocyst-secreting animal or human, or from contaminated food or water [5]. Possible symptoms of cryptosporidiosis include diarrhea, vomiting, nausea, abdominal pain, and fever [6]. Symptoms usually begin a week or two after infection and last a few days to two weeks, but they can persist for up to five weeks [7]. The infection may also occur asymptomatically [8]. In individuals with an immune deficiency, the disease may require hospitalization and can even lead to death [8]. Currently, there is no fully effective drug treatment or vaccine against cryptosporidiosis [7].

In Finland, the incidence of cryptosporidiosis in humans has increased 20-fold in recent years compared to the early 2000s [9]. At the same time, cryptosporidiosis in calves in Finland has also increased [10]. We conducted a case-control study and genotyped *Cryptosporidium* in patient samples to determine the significance of zoonotic *C. parvum* infections in Finland, to identify risk factors for infection, and to confirm the significance of cryptosporidiosis as a work-related disease.

## Methods

### Case-control study

In Finland, laboratory-confirmed *Cryptosporidium* cases are obliged to be reported to the Finnish Infectious Disease Register (FIDR) by the clinical microbiology laboratories [11]. We used FIDR to identify persons with *Cryptosporidium* infections. A case was defined as a person with laboratory-confirmed infection with *Cryptosporidium* species notified to the FIDR between 1 July 2019 and 31 December 2019. New cases were retrieved from FIDR once a week. Cases, and controls picked from the Population Information System (PIS) and matched to cases by age, gender, and hospital district, were sent an invitation letter containing an internet link to respond to a web-based questionnaire. The questionnaire included questions about the demographic and clinical characteristics of the respondents, contact with cattle, and environmental and food exposures. Queries about exposures were limited to within two weeks before the onset of symptoms for cases, and two weeks before answering the questionnaire for controls. The questionnaire could be answered in Finnish or Swedish.

### Microbiological samples and methods

#### Specimens

Clinical microbiological laboratories were asked to send all patient samples that were positive for a *Cryptosporidium* species between 1 July 2019 and 31 December 2019 to the Finnish Food Authority. The clinical microbiological laboratories had analyzed samples with various methods (different polymerase chain reaction (PCR) tests or modified Ziehl-Neelsen staining). All samples from which a *Cryptosporidium* species was originally detected were re-tested by *C. parvum* real-time PCR. In addition, all *C. parvum* negative samples were tested by *C. hominis* real-time PCR [12, 13].

#### Species identification

The processing of feces included cleaning and concentrating the samples using saturated NaCl flotation [14] and an oocyst purification with sodium hypochlorite (0.6% active chlorine) [15]. The releasing of DNA from oocysts included the use of tubes with ceramic beads with a MagNa Lyser (Roche Applied Science, Germany) instrument. A DNA extraction included the use of a QIAamp Mini Kit (Qiagen, Hilden, Germany).

*C. parvum* detection was conducted using a real-time PCR method for rRNA gene of *C. parvum* (CFX96 Touch Real-Time PCR Detection System, Bio-Rad Laboratories, CA, USA) as described previously [12], and commercially available plasmid pUC19 was used as the internal amplification control according to Fricker et al. [13]. Samples in which no *C. parvum* was detected were examined in the same manner for *C. hominis* [12, 13].

#### C. parvum subtyping

*C. parvum* isolates were subtyped by sequence analyses of the nested PCR amplified 60-kDa glycoprotein (gp60) gene [16]. A fragment of the gp60 gene was amplified by a nested PCR protocol according to Alves et al. [17], using primers AL3532 and LX0029 in a secondary PCR [18]. Amplification of secondary products was inspected with gel electrophoresis, and purified with QIAamp MinElute PCR Purification Kit (Qiagen, Hilden, Germany) following the manufacturer’s instructions. The DNA contents of the products were measured with The Qubit® 2.0 Fluorometer (Invitrogen, CA, USA), and samples of sufficient concentration over 0.2 ng/µL were sent directly for sequencing to the DNA Sequencing and Genomics Laboratory (Institute of Biotechnology, University of Helsinki, Finland), or stored at -25 °C prior to shipment. Samples were sequenced with Sanger sequencing in both directions with the primers AL3532 and LX0029. Sequence analyses were conducted with Molecular Evolutionary Genetics Analysis MEGA 7.0.26 software.

The naming of gp60 subtypes was done according to Sulaiman et al. [18]. The categorization of *C. parvum* to families of gp60 subtypes based on differences of the sequence in the non-repeat region of the gp60 gene was done using Basic Local Alignment Search Tool BLAST search, and variation in the trinucleotide repeat region was counted by a researcher.

### Data from the Finnish register of occupational diseases, 2011–2019

Some cases of possible occupational cryptosporidiosis are registered in the Finnish Register of Occupational Diseases (FROD) [19]. In Finland, there are no specific criteria for identifying cryptosporidiosis as an occupational disease. We retrieved occupational cryptosporidiosis cases from FROD between 1 January 2011 and 31 December 2019.

### Statistical analyses

Statistical analyses were conducted using STATA 17.0 software (StataCorp LLC, USA). A univariate analysis was used to calculate odds ratios (OR) with 95% exact confidence intervals (CI) for the exposures, using Chi-square test for P-values. For the analysis the match was broken as controls could not be recruited for 46% of the cases.

Fourteen exposures with the lowest P-value (< 0.16) in the univariate analysis were included in the multivariable analysis, and logistic regression was used as the multivariable model. The explanatory agents were chosen with backward selection using the AIC criterion [20]. To compensate for the match break, clustering was taken into account by using a robust standard error estimator.

## Results

### Species identification and case-control study

In July–December 2019, 291 cryptosporidiosis cases were notified to FIDR from all parts of Finland. The median age was 33 years (1–70 years), and 61% (177/291) were females. The clinical microbiological laboratories sent 411 patient samples from 272 cases (mean 1.5 samples per case, range 1–4) to the Finnish Food Authority. Of patients, 76% (207/272) were infected with *C. parvum*, and 3% (7/272) with *C. hominis*. For the rest, *C. parvum* or *C. hominis* species were not found.

**Fig. 1:**
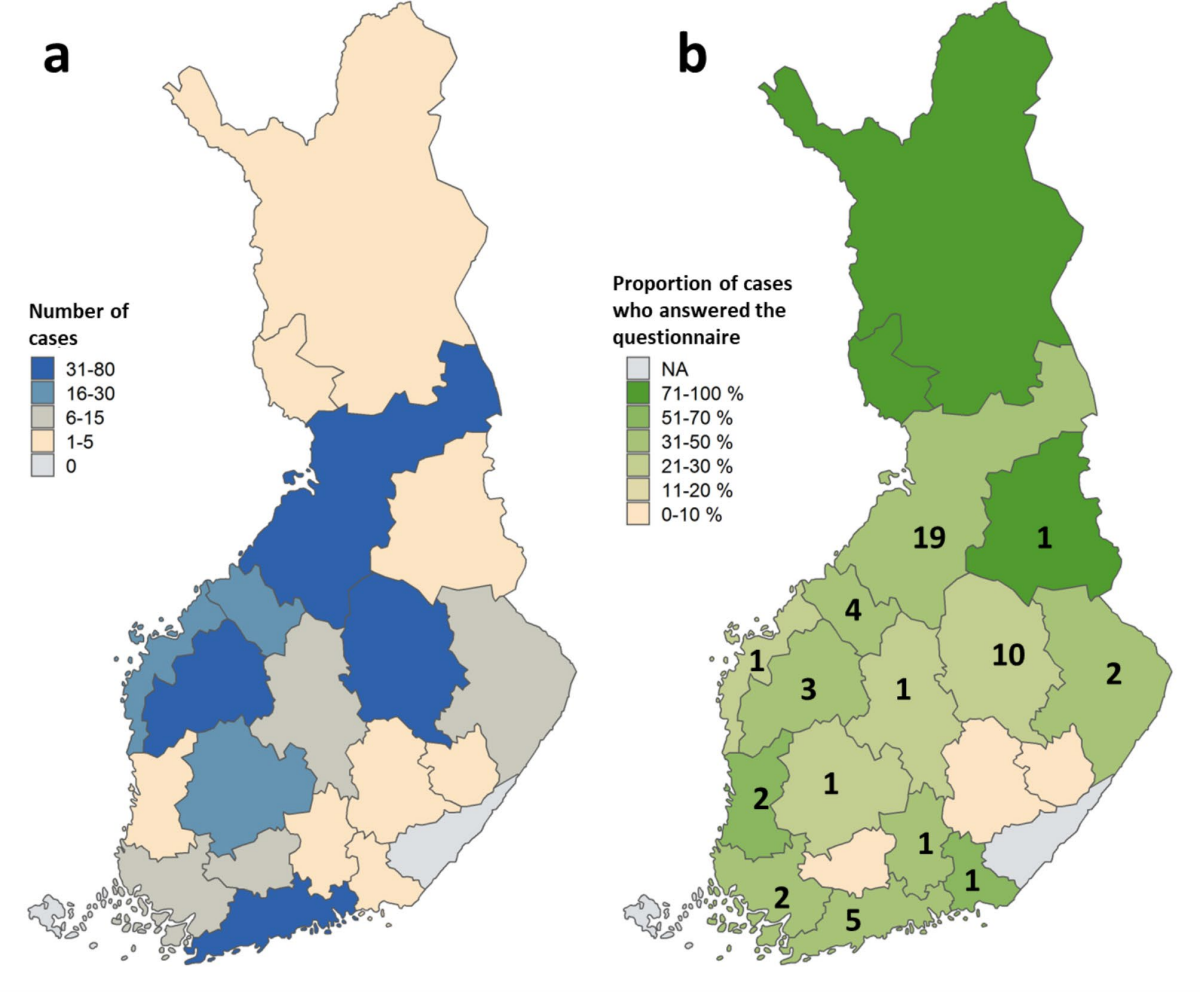
Number of cryptosporidiosis cases invited to answer the questionnaire (a) and the percentage who answered (b) according to hospital district, July–December 2019. Number of cases having cattle contact marked with numbers (b)

From PIS, addresses could be retrieved, and the invitation letter sent to 254 cases (Fig. 1a) and 1,516 controls. The questionnaire response rates were 45% (115/254; Fig. 1b) and 16% (239/1, 516), respectively. From the analysis, 21 controls were excluded due to signs of gastroenteritis.

Samples from 82 cases responding to the questionnaire yielded a positive PCR result at the Finnish Food Authority, all of which were *C. parvum*. These cases were included in the analysis with the 218 controls.

The median age of cases was 37.5 years (5–59 years) and of controls 34 years (1–61 years). In both groups, 68% were female. The most common symptoms were diarrhea (97%), weakness (83%), stomachache (76%), and nausea (76%). The mean duration of symptoms was 12 days (4–26 days). Of the cases, 29% (18/63) received intravenous fluids, and 10% (6/63) were hospitalized at least one night. Traveling abroad in the two weeks before falling ill was reported by 9% (7/82) of cases. They were included in the study since despite traveling, there was also the possibility of having been exposed to cryptosporidia in Finland.

Of the cases, 65% (53/82) had regular cattle contact due to work or studies. Of them, 70% (37/53) were female; the median age was 29 years (5–58 years). Of these cases, 36% (19/53) were from Northern Ostrobothnia and 19% (10/53) from North Savo (Fig. 1b). Five cases from Northern Ostrobothnia and three cases from North Savo could be linked to two cattle farms. Twenty-one cases reported being farmers or farm workers, 10 temporary workers on a farm, 13 students in an animal-related field (including five veterinary students), one was a veterinarian, two reported working in other animal-related jobs (butcher, hoof trimmer), and one was an electrician who had contact with calves while working at a farm.

Most of the cases with regular cattle contact (86%, 44/51) had been on a dairy farm, and 24% (12/51) on a calf rearing unit two weeks prior to falling ill. Of the cases, 70% (37/53) reported visiting farms with diarrhea in cattle, and 97% (36/37) of those reported diarrhea in young calves. Over half of the cases had fed calves with milk (68%) or feed (64%), visited pens of diarrheal calves (53%), or emptied calf pens (53%) (Table 1).

**Table 1:** Work assignments of cases having regular cattle contact due to work or studies (N = 53) in Finland, July–December 2019

Work assignments	Cases
	% (n/N)
Feeding calves with milk	68 (36/53)
Feeding calves with feed	64 (34/53)
Visiting a pen of calves with diarrhea	53 (28/53)
Emptying the calf pen	53 (28/53)
Treating calves with diarrhea	45 (24/53)
Relocating calves	43 (23/53)
Treating sick calves with diseases other than diarrhea	26 (14/53)
Emptying the calving pen	23 (12/53)
Reparation	21 (11/53)
Emptying the isolation pen	19 (10/53)
Washing the calf pen without a pressure washer	15 (8/53)
Dehorning calves	13 (7/53)
Transporting animals	9 (5/53)
Washing the calf pen with a pressure washer	6 (3/53)

Most cases reported washing hands before eating (91%), and when leaving the animal facilities (81%); 54% reported washing hands after treating calves before continuing other work (Table 2). Most cases used protective clothing (98%), clean work shoes or boots (86%), and headwear (87%). A face mask or respirator was usually used by 8% of cases. A personal cellphone was usually used in the animal facilities by 38% of cases, and sometimes by 45%; a protective bag or similar was usually used on the phone by 6%, and sometimes by 20%.

**Table 2:** Working habits and the use of protective equipment at work or studies of cases having regular cattle contact due to work or studies (N = 53) in Finland, July–December 2019

Working habit or protective equipment	Reported conduct of said working habits and usage of said protective equipment, % (n/N)		
	Usually	Sometimes	Never
Washing hands after treating calves (before continuing work)	54 (28/52)	31 (16/52)	15 (8/52)
Washing hands before eating	91 (48/53)	9 (5/53)	0 (0/53)
Washing hands when leaving the animal facilities	81 (43/53)	17 (9/53)	2 (1/53)
Washing shoes/boots after treating calves	79 (41/52)	17 (9/52)	4 (2/52)
Washing shoes/boots when leaving the animal facilities	96 (51/53)	2 (1/53)	2 (1/53)
Washing glasses when leaving the animal facilities	41 (11/27)	26 (7/27)	33 (9/27)
Touching the bovines with bare hands	36 (19/53)	43 (23/53)	21 (11/53)
Hands getting stained by feces while working	17 (9/53)	72 (38/53)	11 (6/53)
Use of personal cellphone in the animal facilities	38 (20/53)	45 (24/53)	17 (9/53)
Use of protective bag or similar on personal cellphone in the animal facilities	6 (3/51)	20 (10/51)	75 (38/51)
Smoking in the animal facilities	0 (0/52)	4 (2/52)	96 (50/52)
Use of snuff in the animal facilities	0 (0/51)	4 (2/51)	96 (49/51)
Chewing gum in the animal facilities	4 (2/51)	16 (8/51)	80 (41/51)
Eating or drinking in the animal facilities	0 (0/51)	20 (10/51)	80 (41/51)
Eating or drinking in a separate room	40 (21/53)	36 (19/53)	25 (13/53)
Protective clothing	98 (52/53)	2 (1/53)	0 (0/53)
Clean work shoes/boots from the farm	86 (44/51)	12 (6/51)	2 (1/51)
Headwear	87 (45/52)	8 (4/52)	6 (3/52)
Disposable gloves	45 (22/49)	37 (18/49)	18 (9/49)
Other protective gloves	54 (27/50)	34 (17/50)	12 (6/50)
Protective gloves when treating calves	56 (29/52)	23 (12/52)	21 (11/21)
Face mask/respirator	8 (4/50)	28 (14/50)	64 (32/50)
Face mask/respirator when treating calves	6 (3/52)	19 (10/52)	75 (39/52)

In the univariate analysis, several exposures were associated with cryptosporidiosis (Table 3). In the multivariable analysis, cryptosporidiosis was associated with having contact with bovines (OR 81, 95% CI 26–251, P < 0.001), having a family member with gastroenteritis (OR 34, 95% CI 6.2–186, P < 0.001), and spending time at one’s own vacation home (OR 15, 95% CI 4.2–54, P < 0.001).

**Table 3:** The results of univariate analysis for different exposure agents. Only 14 exposures with the lowest P-value (< 0.16) are shown

Exposure agent	Cases (N = 82) exposed % (n/N)	Controls (N = 218) exposed % (n/N)	Odds ratio (OR) (95% CI^a^)	P-value
Family member having gastroenteritis	25 (19/77)	4 (8/213)	8.39 (3.27–23.14)	< 0.001
Having contact with bovines	84 (67/80)	12 (26/218)	38.06 (17.60–84.37)	< 0.001
Family member having contact with bovines	42 (33/78)	10 (21/214)	6.74 (3.40–13.41)	< 0.001
Eating or drinking unpasteurized dairy	31 (24/78)	11 (23/210)	3.61 (1.79–7.25)	< 0.001
Dog as pet	57 (47/82)	39 (86/218)	2.06 (1.19–3.57)	0.005
Spending time at own vacation home	16 (13/81)	6 (13/218)	3.10 (1.22–7.41)	0.006
Having a farm near (< 2 km) home	43 (35/81)	27 (57/211)	2.06 (1.16–3.62)	0.008
Blackwater at home: wastewater tank	25 (20/81)	14 (30/216)	2.03 (1.01–4.00)	0.027
Cat as pet	48 (39/82)	34 (74/218)	1.76 (1.02–3.05)	0.030
Greywater at home: wastewater tank	21 (17/81)	12 (26/217)	1.95 (0.93–4.01)	0.049
Other contact with cats	44 (36/82)	33 (72/218)	1.59 (0.91–2.75)	0.080
Washing root vegetables purchased from farmer	92 (66/72)	84 (179/213)	2.09 (0.81–6.36)	0.107
Blackwater at home: other	23 (19/81)	16 (34/216)	1.64 (0.82–3.21)	0.122
Greywater at home: other	26 (21/81)	18 (40/217)	1.55 (0.80–2.94)	0.154

^a^ = Confidence interval

### Gp60-subtyping of patient isolates

**Fig. 2:**
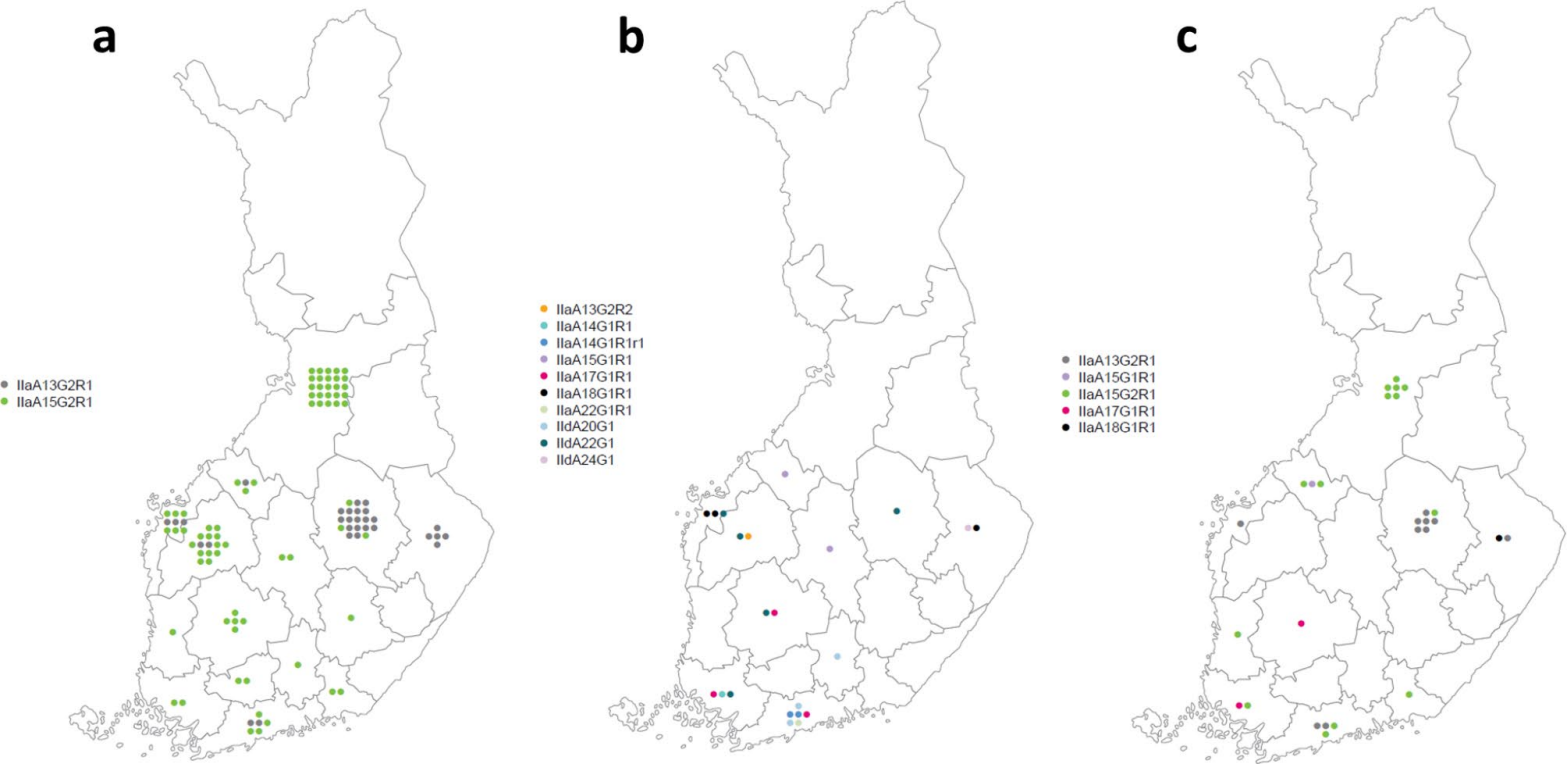
Distribution of *C. parvum* gp60 subtypes according to hospital district in humans in Finland, July–December 2019 (a: two most common subtypes detected; b: other detected subtypes; c: subtypes of cases having cattle contact)

A total of 141 *C. parvum* samples were subtyped, and 124 samples yielded 13 gp60 subtypes (Fig. 2); 17 samples yielded no result. The gp60 subtypes detected belonged to two subtype families: 91% (113/124) to subtype family IIa, and the rest to subtype family IId. Two subtypes, IIaA15G2R1 (56%, 70/124) and IIaA13G2R1 (24%, 30/124), were the most common (Fig. 2a). The other gp60 subtypes detected were IIaA13G2R2, IIaA14G1R1, IIaA14G1R1r1, IIaA15G1R1, IIaA17G1R1, IIaA18G1R1, IIaA22G1R1, IIdA20G1, IIdA21G1, IIdA22G1, and IIdA24G1 (Fig. 2b). One representative sequence of each of the 13 different gp60 subtypes was deposited in the GenBank database under accession numbers OQ942864–OQ942876. In cases with cattle contact, subtypes IIaA15G2R1 (50%, 14/28) and IIaA13G2R1 (36%, 10/28) were most commonly identified (Fig. 2c). Of five cases from Northern Ostrobothnia linked to the same farm, two samples could be subtyped, both being IIaA15G2R1. Of three cases from North Savo linked to the same farm, two samples were subtyped as IIaA13G2R1.

### Data from the Finnish register of occupational diseases, 2011–2019

In 2011–2019, 68 recognized (median 1, range 0–46/ year) cases of occupational cryptosporidiosis were registered in FROD.

## Discussion

Zoonotic *C. parvum* was the most common *Cryptosporidium* species detected, and cryptosporidiosis in Finland was strongly associated with cattle contact. The highest cattle densities in Finland are in the regions of Ostrobothnia and North Savo [21], where also most cryptosporidiosis cases were observed during the study period of July–December 2019 (Fig. 1a). Considering the high number of cryptosporidiosis cases in the regions with the highest cattle densities in Finland, it is likely that contact with cattle is a major risk factor for zoonotic *Cryptosporidium* infections in humans in those areas. This is also reflected in the geographical distribution of *C. parvum* cases having regular contacts with cattle due to work or studies (Fig. 1b).

The majority of the cryptosporidiosis cases having regular contact with cattle had been on a dairy farm prior to illness. Almost all of them worked on farms, were students in an animal-related field, or worked in other animal-related jobs (veterinarian, butcher, hoof trimmer, electrician who had worked on a cattle farm). Dairy farms and calf rearing units usually have calves under six weeks of age that may be infected with *C. parvum* [4] and may pose a risk of *C. parvum* infection to persons handling the calves. Most of the cattle-related cryptosporidiosis cases in our study reported participating in tasks related to calf care, the cleaning of pens, and visiting the pens of calves with diarrhea. People working on farms with cattle such as farmers, farm workers, veterinarians, and veterinary students have also been shown to have an occupational health risk of cryptosporidiosis in previous studies [22–25]. Outbreaks of cryptosporidiosis have been reported in veterinary students in several European countries and in the United States [22–25].

As shown in our study and in others, cryptosporidiosis can be linked to working or living on cattle farms or visiting one. Based on infectivity, *C. parvum* is classified in risk group 2 in EU directives on the protection of workers from risks related to exposure to biological agents at work [26, 27]. We assess the cryptosporidiosis risk level to be moderate to high. The risk assessment was done descriptively by taking the following factors into account: The probability of exposure is likely because *C. parvum* is found in cattle in most farms, and workers can be exposed to *C. parvum* daily in barns. The consequences of infection may be moderate because the symptoms of cryptosporidiosis usually last for about 10 days, and 10% of those affected may be hospitalized. The recognition of cryptosporidiosis as an occupational disease enables those affected to receive appropriate compensation for their illness and promotes improvements in occupational safety in cattle-related jobs. To treat those infected equally, there is a need to agree on specific criteria for identifying cryptosporidiosis as an occupational disease.

Since 2016, *C. parvum* has become more common on cattle farms [10], and the reporting of occupational cryptosporidiosis has increased. In the same period, the number of suspected cases of occupational disease has also increased in FROD, but there have been problems in proving their work-related origin. The investigation of the origin of the disease may have started later than cryptosporidiosis could have been found in cattle, or livestock samples were not available. On the other hand, the employee may have worked with several herds before becoming ill, and it has not been possible to verify where the exposure to *Cryptosporidium* took place. The reporting of cryptosporidiosis to FIDR has increased since 2016, partly due to the introduction of PCR diagnostics [9]. The annual number of occupational cryptosporidiosis reports is in line with the results of our six-month questionnaire study, in which 35 work-related infections were identified compared to 46 reported in FROD in 2019.

Farm work-related risk factors associated with *C. parvum* infection include contact with young cattle or calves with diarrhea, ingestion of food or water contaminated with livestock manure, washing hands without soap, and protective equipment stained with feces [6, 23, 25]. On the farm, infection may also be obtained without direct animal contact via contaminated objects in the working environment [23]. Oocysts may be transferred from contaminated equipment such as cellphone to hands. In our study, the use of a personal cellphone in animal facilities was very common, and a protective phone bag was rarely used. We recommend the use of a disposable or washable protective bag on a cellphone when in animal facilities. To prevent oocysts from spreading, cellphones without a protective bag could be handled with clean hands only.

Key practices for protection against cryptosporidiosis at the farm level include good hand hygiene, the use of personal protective equipment (PPE) when in contact with calves, and adequate changing facilities to remove protective equipment when leaving the barn [22]. The infectious dose of *Cryptosporidium* is small: only a dozen protozoan oocysts are required to cause infection [8]. Thus, *Cryptosporidium* is highly infectious due to high numbers of oocysts excreted in feces [6]. Furthermore, oocysts excreted in feces are persistent in the environment, especially in humid conditions [28], and are highly resistant to many disinfectants [29]. Occupational exposure should be reduced by infection control procedures, which can be challenging due to the aforementioned features of the *Cryptosporidium* oocysts. Several studies highlight the importance of hand hygiene, such as hand washing with soap, in the protection against this feco-orally transmitted infection [22, 23, 25]. In our study, respondents reported washing their hands moderately well. The majority reported washing their hands before eating as well as when leaving the animal facilities. About half of the respondents reported washing their hands after treating calves before continuing other work. Taking care of hygiene is of paramount importance in the prevention of cryptosporidiosis, and personal hygiene practices could be improved by providing appropriate washing facilities in barns.

The appropriate use of PPE when working with cattle, and especially with calves, is important [22, 25], as well as proper decontamination of PPE (e.g., protective clothing) after leaving animal facilities [22, 23]. Most of the respondents in our study reported wearing protective clothing, clean work shoes or boots, and headwear, as well as washing boots after leaving the animal facilities, and after treating calves. However, only half of the respondents reported wearing protective gloves when treating calves, and only a few used a face mask or respirator. To facilitate maintaining good hand hygiene, the use of protective gloves when handling calves is recommended. Due to feco-oral transmission, a respirator or face mask could be used to protect against fecal splashes when working with calves, especially when handling calves with diarrhea.

Sequence analysis of gp60 gene is a widely used method for elucidating genetic differences in *Cryptosporidium* and subtyping *Cryptosporidium* species to understand the transmission of infection in humans and animals [5]. The same gp60 subtypes of *C. parvum* have been reported in calves and humans with cryptosporidiosis [5, 23, 25]. Studying the *Cryptosporidium* species that cause cryptosporidiosis in humans, determining subspecies using molecular methods such as gp60 subtyping, and identifying the sources of infection is paramount in order to understand the transmission of infections and the epidemiology of human cryptosporidiosis, and to direct infection prevention efforts correctly.

Of all the patient samples examined, *C. parvum* accounted for 76% and *C. hominis* for 3%. The high proportion of cryptosporidiosis cases caused by *C. parvum* when compared to *C. hominis* has also been seen in previous studies from industrialized countries, such as Sweden, Ireland, France, the Netherlands, and Canada [30–34]. In many of those countries, *C. hominis* have caused around a quarter of infections, showing that although zoonotic transmission is important, anthroponotic transmission should not be disregarded. In Sweden, however, domestic infections were primarily caused by *C. parvum* (84%) and only to lesser extent by *C. hominis* (3%) [32], which corresponds with our findings. As in Finland, the incidence of cryptosporidiosis in humans in Sweden has also increased in recent years [35].

*C. parvum* gp60 subtypes detected in patient samples belonged to zoonotic subtype families IIa and IId, which occur in both humans and ruminants. Subtype family IIa is a common subtype family infecting calves, and IId is also found in calves [5, 6]. In humans, *C. parvum* subtype family IIa is a common cause of infections in industrialized countries, and IId is found to a lesser extent in Europe [36]. The most common *C. parvum* gp60 subtype in the patient samples examined was IIaA15G2R1, found particularly in western Finland. The second most common gp60 subtype was IIaA13G2R1, which was concentrated more in eastern Finland. Together, these two subtypes represented the majority of the gp60 subtypes found in patient samples. IIaA15G2R1 is a prevalent subtype found in calves in many countries around the world [5] and is also a prevalent subtype in humans in most industrialized countries [36]. In addition, IIaA13G2R1 has been found in both calves [37–39] and humans [32, 40]. In 2018, subtypes IIaA15G2R1, IIaA13G2R1, IIaA15G1R1, and IIaA18G1R1 were identified in an outbreak investigation in Finland [9]. The same subtypes were also found in this study. In Finland, the high proportion of human infections caused by *C. parvum*, combined with the same subtypes commonly found in calves, probably illustrates the burden of disease attributable to zoonotic transmission.

In this study, cryptosporidiosis was linked to cattle contact, a family member suffering from gastroenteritis, and spending time at one’s own vacation home. Contact with cattle, visiting or living on a farm, and household person-to-person transmission have also explained cryptosporidiosis in previous studies [33, 41, 42]. In Finland, people commonly spend their holidays in vacation homes, usually located in the countryside, which makes visits to livestock farms possible. Some vacation homes also have poorer facilities for maintaining good hygiene in practices such as cooking or hand washing. Also, contaminated water may be the source of infection at vacation homes. Only a few cases had traveled abroad before they fell ill; thus, traveling does not explain the increase in cryptosporidiosis cases in Finland.

### Strengths and limitations

To this extent, *Cryptosporidium* species and exposures associated with cryptosporidiosis were analyzed for the first time in Finland in this study. From clinical laboratories, we received samples from almost all cryptosporidiosis cases in July–December 2019, which enabled us to get an overview of *Cryptosporidium* species distribution in Finland. However, this study was restricted to species *C. parvum* and *C. hominis* indicating that the species distribution in Finland should be investigated further. A longer study period would be needed to detect possible seasonal variations.

People with cattle contact may have been more likely to respond to the questionnaire compared to other individuals with cryptosporidiosis. The increased risk of zoonotic *Cryptosporidium* infection on cattle farms has been discussed in Finland in recent years. Therefore, people working with livestock may have been more interested in the topic than the rest of the population. Furthermore, patients with symptoms of cryptosporidiosis, who have been in contact with calves, may seek health care more readily.

In case-control studies, recollection bias is always possible [43]. However, participants were asked to complete the questionnaire soon after illness, and most of the questions concerned recurring or outstanding exposures, which are usually easier to remember.

## Conclusions

Cryptosporidiosis should be recognized as a significant occupational disease among persons working with livestock in Finland. Health care professionals should be aware that the risk of exposure to *Cryptosporidium* and the development of cryptosporidiosis is moderate to high on Finnish cattle farms today. In order to detect cryptosporidiosis outbreaks and find infection sources, *Cryptosporidium* species should be routinely determined in patient samples, and subtyping performed when needed. We recommend that persons working on or visiting cattle farms should (a) be informed about the risk of cryptosporidiosis, especially in diarrheal calves; (b) pay more attention to hygiene practices, such as thorough hand hygiene; and (c) use protective equipment, especially when in contact with calves, to avoid infections. For people working on cattle farms and farm visitors, detailed guidance on hygiene practices to reduce the risk of contracting cryptosporidiosis is needed.

## Data Availability

The raw/processed data analyzed in the study cannot be shared due to European General Data Protection Regulation.

## Declarations

### Prior publication

Data included in this article have previously been published in the Abstract Book of the 3rd Annual Scientific Meeting of The One Health EJP, Copenhagen, Denmark and online, June 9th − 11th 2021.
